# Hydrogel Microarray for Bioanalytical Applications: Preliminary Study on Material Properties

**DOI:** 10.3390/ma18133118

**Published:** 2025-07-01

**Authors:** Weronika Kieres, Sonia Kudłacik-Kramarczyk, Joanna Marczyk, Celina Ziejewska, Anna Drabczyk, Robert P. Socha, Marcel Krzan

**Affiliations:** 1 Jerzy Haber Institute of Catalysis and Surface Chemistry, Polish Academy of Sciences, 8 Niezapominajek St., 30-239 Krakow, Poland; weronika.kieres@ikifp.edu.pl (W.K.); marcel.krzan@ikifp.edu.pl (M.K.); 2 Faculty of Mechanical Engineering, Cracow University of Technology, 37 Jana Pawła II Av., 31-864 Krakow, Poland; joanna.marczyk@pk.edu.pl; 3 CBRTP SA Research and Development Center of Technology for Industry, Zygmunta Modzelewskiego 77 St., 02-679 Warszawa, Poland; anna.drabczyk@cbrtp.pl (A.D.); robert.socha@cbrtp.pl (R.P.S.)

**Keywords:** hydrogels, UV photopolymerization, sodium alginate, betaine, gum arabic, swelling ratio, FT-IR spectroscopy, contact angle, SEM, biomedical materials

## Abstract

The aim of this study was to develop and characterize UV-crosslinked hydrogel matrices based on polyethylene glycol diacrylate (PEGDA), gum arabic, betaine, and sodium alginate for potential bioanalytical applications. Various physicochemical analyses were performed, including pre-polymerization emulsion stability (Multiscan), FT-IR spectroscopy, swelling behavior in physiological buffers, pH monitoring, contact angle measurements, and morphological assessment via SEM and optical microscopy. The results demonstrated that both alginate content and UV exposure time significantly influence the structural and functional properties of the hydrogels. The highest swelling ratio (2.32 g/g) was observed for the formulation containing 5% sodium alginate polymerized for 5 min (5SA_5), though this sample showed mechanical fragmentation during incubation. In contrast, the most balanced performance was achieved for the 10SA_15 formulation, which maintained structural integrity and exhibited a swelling ratio of 1.92 g/g after 9 days. The contact angle analysis revealed a surface hydrophilicity range from 50° to 100°, with the lowest angle (50°) recorded for 10SA_5, indicating high surface wettability. These findings confirm the suitability of such hydrogels for biomedical applications, particularly as absorbent, stable platforms for drug delivery or wound healing.

## 1. Introduction

Hydrogels are crosslinked macromolecular hydrophilic polymers forming three-dimensional hydrophilic structures, which are used in agriculture, the food industry, sanitary materials, biomedical applications, including tissue engineering, drug delivery systems, dealing with injuries, regenerative medicine, as well as biosensors [1,2,3,4]. Although they are not soluble in aqueous solutions, due to their structure, they can absorb and retain water in quantities of up to 99% without losing their shape [5,6]. Therefore, they can accept bioactive structures, for instance, proteins, DNA and RNA acids, or another biomolecule [7]. In general, hydrogels may be produced from natural as well as synthetic polymers; however, application of natural polymers in the synthesis of hydrogels is becoming more and more popular in the scientific community, particularly due to their biocompatibility and non-toxicity. Therefore, researchers in their works have investigated hydrogels containing naturally occurring polymers, such as cellulose [8], chitosan [9], pectin [10], lignin [11], alginate [12], and agar and carrageenan [13].

One of them is sodium alginate, a natural polysaccharide characterized by its molecular structure, which contains α-L-guluronic and β-D-mannuronic acids connected by 1,4-glycosidic bonds. It is well-known due to its great biodegradability, non-toxicity, oxygen permeability, hydrophilicity, processing ability, environmental compliance, gelation potential, and speed, as well as low price [14,15,16]. Alginate in combination with divalent cations, including Ca^2+^ or Zn^2+^, which constitute cross connections for functional groups of alginate bonds, creates gel structures [17]. In addition, it is the most commonly used material in tissue engineering, and according to the U.S. Food and Drug Administration it is a safe product [18].

Microarrays are widespread bioanalytical devices composed of hydrogel parts, usually appearing as spots or blobs, containing immobilized probes or biomolecules, mainly DNA (DNA chips) and proteins, which are located on a substrate. Their current rapid development and popularity stem from an opportunity to provide fast, accurate, simultaneous, and reliable detection of biological materials, as well as detection of their quantity. This technology is used in medicine, molecular biology, and clinical laboratories, enabling rapid diagnosis and drug discovery and screening [19,20,21,22,23]. Substrates made of silicon or glass are applied conventionally, but microarrays with hydrogel materials, due to their properties, have gained significant interest in recent years [24,25].

Microneedle arrays, also called microarray patches, are an example of a different type of technology due to the possibility of transdermal drug delivery, vaccines, or even real-time monitoring of glucose, C-reactive protein (CRP), or cytokine levels. These devices are wearable directly on the skin, the largest organ covering the entire outer surface of the human body, which constitutes a huge advantage. Furthermore, the skin fulfils many functions simultaneously, including protecting cells and tissues against loss of electrolytes, mechanical and chemical damage, UV radiation, hazardous substances, infectious diseases, and temperature changes [26,27,28,29].

Datar et al. [30] have described miniaturized three-dimensional cell cultures in hydrogels using cell printing techniques. Cellular microdrops are placed in hydrogel on the surfaces of slides or plastic chips using microarray spotters. In this way, the prepared structures are cultured in appropriate media to produce three-dimensional, cell-surrounded droplets used in various bioassays. This helps control cellular microenvironments and the difficulties associated with obtaining clear images of cells due to problems with focus and material transparency. Wales at al. [31] reported the first example of 3D printing a mixture from poly(octamethylene maleate citrate) (POMaC) and poly(ethylene glycol diacrylate) (PEGDA) in various proportions. Qualitative evaluation of the printed parts showed the possibility of creating complex shapes. The addition of PEGDA did not negatively affect the degradability and biocompatibility of the material compared to pure POMaC, while significantly improving the adhesion strength (especially at the 90/10 ratio). PEGDA has been proven to enable the 3D printing of complex POMaC molds, and to tune and optimize the physical properties of the printed parts by changing their composition. Such customizable, 3D-printed biomaterials based on POMaC and PEGDA have the potential to be used as functional tissue scaffolds. Andersson et al. [32] analyzed a hydrogel biosensor matrix suitable as a template for evaluating specific and non-specific binding phenomena for protein microarray sensors. The matrix was based on a copolymer of poly(ethylene glycol) methacrylate and 2-hydroxyethylmethacrylate, produced by photoinitiated UV polymerization. Analysis using surface plasmon resonance (iSPR) imaging was applied. The results confirmed that the gradient hydrogel matrix is suitable for rapid testing and optimization of binding conditions. The performance of this matrix was evaluated in situ by studying biomolecular interactions between human serum albumin (HSA) and its antibody (aHSA) in spots containing different proportions of HEMA and PEG_10_MA. It was shown that the matrix containing 25% PEG_10_MA effectively prevented non-specific fibrinogen (HFib) binding. At the same time, the ability of carboxyl groups introduced into the hydrogel to immobilize HSA increased with increasing HEMA content, indicating that a content of 75% HEMA may be optimal for practical application. Then, Pikula et al. [33] presented a one-step printed hydrogel microarray integrating long DNA chains for discriminative and size-specific sensing of nucleic acids. Due to the controlled porosity of the hydrogel and immobilization of RCA products, more than 65% signal retention and high structural stability are achieved. The system enables the discrimination of short oligonucleotides (≤27 nucleotides) and sensitive detection without the need for signal amplification, achieving a signal-to-noise ratio of >10 in just 30 min. The anti-adsorption properties of the hydrogel promote specific hybridization in complex samples, which is particularly important for short oligonucleotides (~10 nt). Due to their ease of production and high efficiency, CAR-based hydrogel microarrays represent a promising tool for the rapid, parallel, and cost-effective detection of short DNA/RNA biomarkers and ligands, a key challenge in modern molecular diagnostics and bioanalytical screening.

Until now, the effect of sodium alginate as well as UV polymerization time on the physicochemical properties of hydrogel microarray has not been fully characterized. Although many researchers have conducted experiments regarding microarrays, issues regarding the physicochemical properties of hydrogels applied in these devices still require further study to fill the existing research gap. Furthermore, there is still a lack of standards concerning optimal parameters of hydrogels in terms of bioanalysis, such as stability, or wettability of the surface. Therefore, in the presented study, cross-linked hydrogel microarrays for bioanalytical applications were synthesized via fast UV photopolymerization using sodium betaine, gum arabic, crosslinking agent, and photoinitiator. The impact of sodium betaine and the UV polymerization time was explored in detail using a broad spectrum of research techniques, such as Multiscan analysis to assess emulsion stability before photopolymerization, pH examination realized during hydrogel incubation, as well as determining the swelling behavior of materials. Furthermore, the morphology of synthesized biomaterials was observed using a scanning electron microscope (SEM) and a digital one. The results presented in this work involve the early stage of microarray design, revealing the properties of hydrogels before they are applied in bioanalytical applications. This approach provides a full understanding of the impact of the physicochemical characteristics of hydrogels on the quality of subsequently obtained microarrays.

## 2. Materials and Methods

### 2.1. Materials

Poly(ethylene glycol) diacrylate (PEGDA, average molecular weight 575 g/mol), sodium alginate (natriumalginat, CAS-No: 9005-38-3), gum arabic (extracted from acacia tree, branched polysaccharide, CAS-No: 9000-01-5), betaine (C_5_H_11_NO_2_, M = 117.15 g/mol, CAS-No: 107-43-7, ≥98%), and 2-hydroxy-2-methylpropiophenone (photoinitiator, 97%) were purchased from Sigma Aldrich (Saint Louis, MO, USA). All reagents were used as received without further purification. Ultrapure water was used for the preparation of all hydrogel formulations.

### 2.2. Synthesis of Hydrogel Dressings Under the Influence of UV Radiation

Hydrogels were synthesized via photopolymerization using UV radiation. This method was chosen due to its short reaction time, low energy requirements, lack of by-product formation, and the additional advantage of material sterilization during polymerization—a key factor in the context of potential biomedical applications.

The formulation process began with the preparation of aqueous stock solutions: 5% (*w*/*v*) betaine, 5% (*w*/*v*) gum arabic, and 2.5% (*w*/*v*) sodium alginate. Each compound was dissolved in ultrapure water under continuous magnetic stirring for 1 h at 35 °C, until full dissolution was achieved. The solutions were not filtered. All stock solutions were freshly prepared immediately prior to hydrogel synthesis. For each sample, components were combined in the order presented in Table 1: betaine solution, gum arabic solution, sodium alginate solution (if applicable), PEGDA as the crosslinking agent, and 2-hydroxy-2-methylpropiophenone as the photoinitiator.

After mixing the appropriate volumes of reagents, the resulting homogeneous mixtures were poured into reaction vessels. Photopolymerization was carried out using a UV lamp (EMITA VP-60, Famed, Łódź, Poland) with the following parameters: power—120 W, applied wavelength—320 nm. Exposure time was set to either 5 or 15 min, depending on the formulation, as detailed in Table 1.

**Scheme 1:**
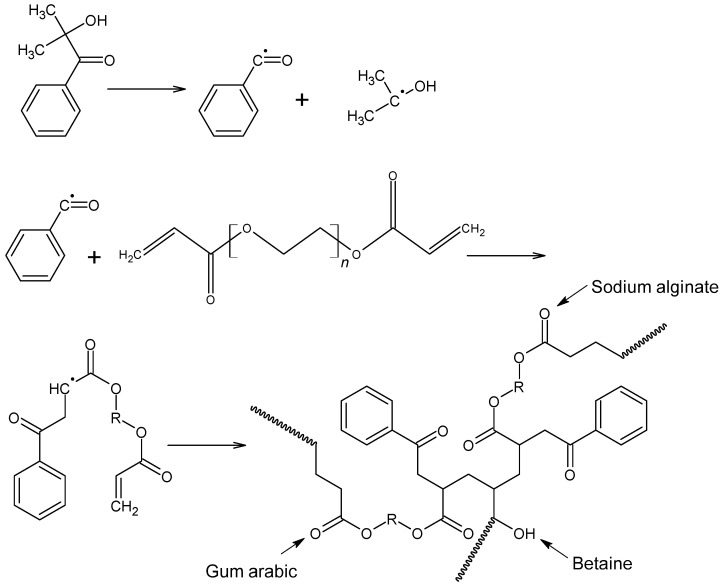
Proposed mechanism of UV-induced radical polymerization of PEGDA in the presence of sodium alginate, gum arabic, and betaine (the dot represents a free radical species formed during the reaction.).

The simplified chemical reaction scheme of the UV-initiated photopolymerization process is presented in Scheme 1.

Under UV irradiation, the photoinitiator (2-hydroxy-2-methylpropiophenone) undergoes homolytic cleavage, generating reactive radicals. These radicals initiate the polymerization of the acrylate end groups of PEGDA, forming a covalently crosslinked network. While PEGDA provides the primary scaffold through covalent bonding, the incorporated additives—sodium alginate, gum arabic, and betaine—do not chemically react but interact physically with the forming polymer chains. These interactions occur predominantly through hydrogen bonding (via hydroxyl and carboxyl groups) and electrostatic forces (in the case of zwitterionic betaine), contributing to the hydrogel’s swelling capacity, porosity, and structural stability.

**Figure 1:**
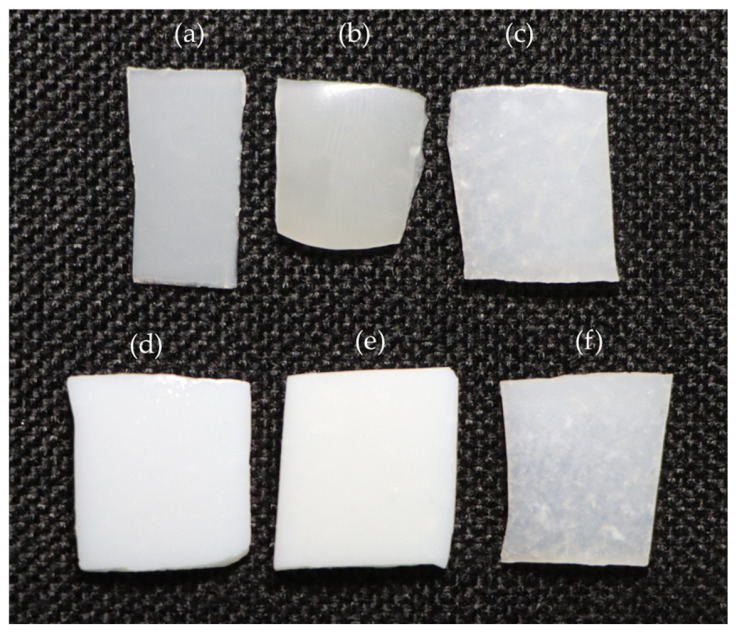
Visual comparison of hydrogel samples immediately after photopolymerization. From top left to bottom right: (**a**) 0SA_5, (**b**) 0SA_15, (**c**) 5SA_5, (**d**) 5SA_15, (**e**) 10SA_5, and (**f**) 10SA_15. Samples differ in sodium alginate content and UV polymerization time, as specified in Table 1.

The hydrogel formulations listed in Table 1 were visually distinct following synthesis. Selected samples were compared to illustrate the influence of sodium alginate concentration on the appearance and uniformity of the materials (see Figure 1).

The visual appearance of the hydrogel samples after photopolymerization reveals clear differences in opacity and surface uniformity depending on the alginate concentration and UV curing time. Samples (a) and (b), lacking sodium alginate, remain relatively translucent with smooth and minimally structured surfaces. In contrast, sample (c), which contains 5 mL of alginate, appears more opaque and exhibits visible heterogeneity, suggesting an increase in internal porosity. Sample (d), cured for a longer time with the same alginate content, presents a more uniform appearance. Samples (e) and (f), formulated with 10 mL of alginate, are the most opaque. Notably, sample (e) appears visually smoother and more homogeneous, whereas sample (f) displays minor surface irregularities, possibly indicating differences in network formation or gel uniformity. These variations highlight the influence of formulation parameters on the macro-scale structure and consistency of the hydrogels.

### 2.3. Emulsion Stability Before Photopolymerization: Multiscan Analysis

The stability of prepolymer mixtures was assessed using a Multiscan MS20 system (DataPhysics Instruments GmbH, Filderstadt, Germany). The primary objective of this analysis was to evaluate the homogeneity of the formulations prior to the photopolymerization process and to detect any potential phase separation, which could adversely affect the final properties of the resulting hydrogel materials.

Measurements were conducted immediately after mixing the components and then repeated after 24 h of storage at room temperature. This allowed for the identification of any time-dependent destabilization phenomena such as sedimentation, creaming, or phase boundary formation.

### 2.4. FT-IR Spectroscopy of Hydrogels: Post-Synthesis and Post-Incubation Analysis

The effect of incubation in simulated physiological fluids on the chemical structure of the hydrogel materials was evaluated using Fourier-transform infrared spectroscopy (FT-IR) with the attenuated total reflectance (ATR) technique. Spectra were recorded using a Thermo Scientific Nicolet iS5 spectrometer (Waltham, MA, USA) equipped with a diamond ATR crystal.

Measurements were performed in the spectral range of 4000–500 cm^−1^, with a resolution of 0.4 cm^−1^ and 32 scans per sample. All spectra were acquired at room temperature. The obtained spectra were analyzed to identify characteristic functional groups present in the hydrogel compositions and to detect potential chemical changes occurring as a result of incubation.

### 2.5. Swelling Behavior of the Hydrogel Matrix

The ability of the hydrogel matrices to absorb liquids was evaluated in various aqueous environments. The tested media included phosphate buffer, acetate buffer, 0.9% potassium chloride solution, 1 M barium chloride dihydrate solution, and distilled water, which served as the reference.

Each dry hydrogel sample, weighing approximately 1.0 g, was immersed in 50 mL of the selected medium. The swelling behavior was monitored at three time points: after 24 h, 48 h, and 216 h (9 days) of incubation. At each time point, the sample was removed from the solution, gently blotted to remove excess surface liquid, and weighed. After weighing, the sample was returned to the same medium for further incubation.

Swelling capacity was expressed as the ratio of the mass increase relative to the initial dry weight. This allowed for the comparison of swelling kinetics across different media and formulations. All measurements were performed in triplicate (*n* = 3), and the results are reported as mean values with standard deviations.

### 2.6. pH-Dependent Behavior During Hydrogel Incubation

To assess the chemical stability of the hydrogels and their potential impact on the surrounding environment, samples were incubated in selected simulated physiological fluids for eight days. The tested media included phosphate buffer, acetate buffer, 0.9% potassium chloride solution, and 1 M barium chloride dihydrate solution.

Each hydrogel sample was immersed in 50 mL of the respective solution and incubated at 37 °C to mimic physiological conditions. Throughout the incubation period, the pH of each solution was monitored using a CX-701 pH meter (Elmetron, Zabrze, Poland) to detect any shifts that might indicate material degradation or ion exchange processes. Measurements were performed at regular intervals to track temporal pH changes.

### 2.7. Wettability Assessment of the Hydrogel Surface

The wettability of the developed hydrogels was investigated to assess how the UV polymerization time affects the surface interaction with liquids. Measurements were conducted using an optical contact angle goniometer (Drop Shape Analyzer Krüss DSA100M, Hamburg, Germany).

Before the analysis, hydrogel discs with a diameter of approximately 1 cm were dried at 40 °C until a constant mass was achieved to ensure uniform surface conditions. Once dried, each sample was positioned on the measurement stage, and a droplet of distilled water was carefully applied to the flattest surface area. The contact angle was recorded after reaching equilibrium.

The experiment was performed at room temperature and repeated 3 times per formulation to ensure measurement reproducibility.

### 2.8. SEM Characterization of Hydrogel Network Structure

The surface morphology of the hydrogel materials was examined using a scanning electron microscope (Phenom Pharos, Thermo Fisher Scientific, Waltham, MA, USA). Rectangular hydrogel samples (1.0 × 0.1 × 0.1 cm) were sputter-coated with a thin layer of copper to enhance surface conductivity and ensure high-quality imaging. The coating process was carried out using a Denton Vacuum DESK IV Cold Sputter system (Denton Vacuum, Moorestown, NJ, USA).

All observations were performed at room temperature. The SEM images were used to evaluate the surface architecture of the freshly synthesized hydrogels and to compare the influence of formulation differences on the development of the porous polymer network.

### 2.9. Surface Morphology by Optical Microscopy

A 4K high-precision digital microscope VHX-D500/D510 (Keyence, Osaka, Japan) was employed to analyze the surface morphology of the hydrogel samples in order to assess the influence of chemical composition and polymerization time on the structural organization and homogeneity of the formed networks. Imaging was performed at ambient temperature.

## 3. Results and Discussion

### 3.1. Results of the Multiscan Analysis of Emulsion Stability

**Figure 2:**
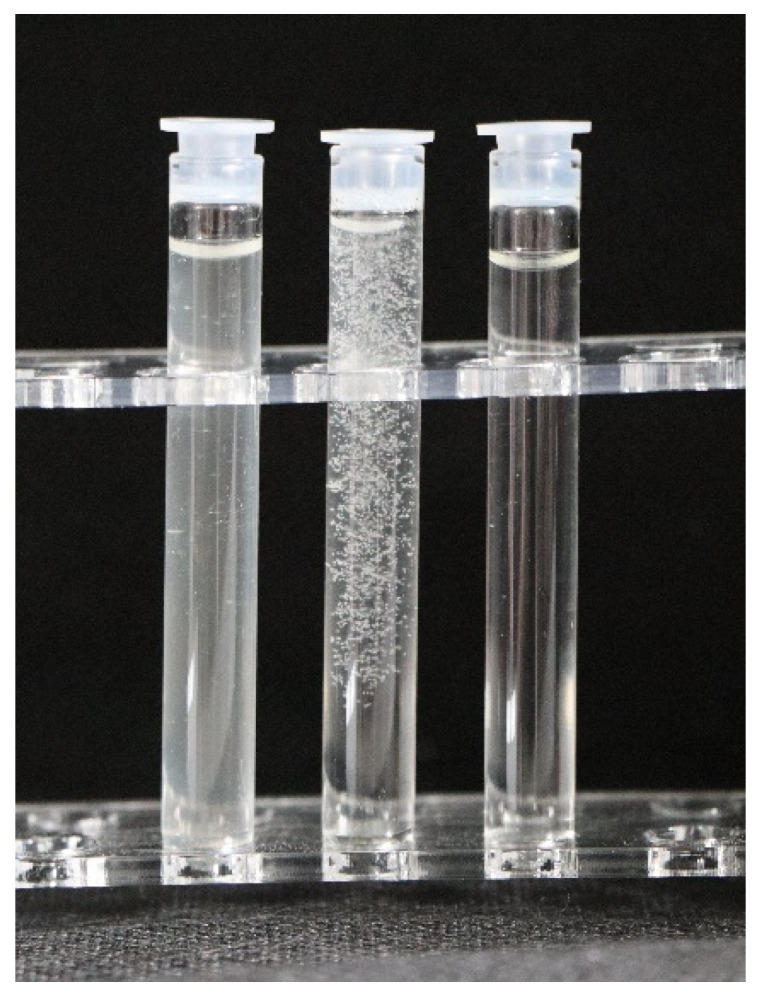
Visual appearance of prepolymer mixtures before photopolymerization. From left to right: Sample 0SA (no sodium alginate), Sample 5SA (with 5 mL of 2.5% sodium alginate), and Sample 10SA (with 10 mL of 2.5% sodium alginate), representing increasing concentrations of sodium alginate in the formulations.

The uniformity and the phase stability of prepolymer compositions were assessed prior to UV curing. The results presented below provide insight into the potential sedimentation or phase separation in different hydrogel formulations. Figure 2 presents the visual differences in phase stability among the formulations with varying sodium alginate content.

The observed differences in the appearance of the samples are attributed to the influence of sodium alginate content on the physicochemical properties of the prepolymer mixtures. Sample 0SA, which did not contain alginate, appeared turbid, possibly due to the absence of the stabilizing effect provided by the polysaccharide and insufficient dispersion of components in the aqueous matrix. Sample 5SA, containing a moderate amount of alginate (5 mL), exhibited a large number of air bubbles and visible heterogeneity, which may indicate inadequate degassing or vigorous foaming during mixing. In contrast, Sample 10SA, with the highest alginate concentration (10 mL), was the most transparent. This effect could be attributed to the increased viscosity of the system, which may have suppressed air bubble formation and retention, while also promoting colloidal stability. Additionally, the higher alginate content likely enhanced the compatibility of components, resulting in improved phase uniformity.

**Figure 3:**
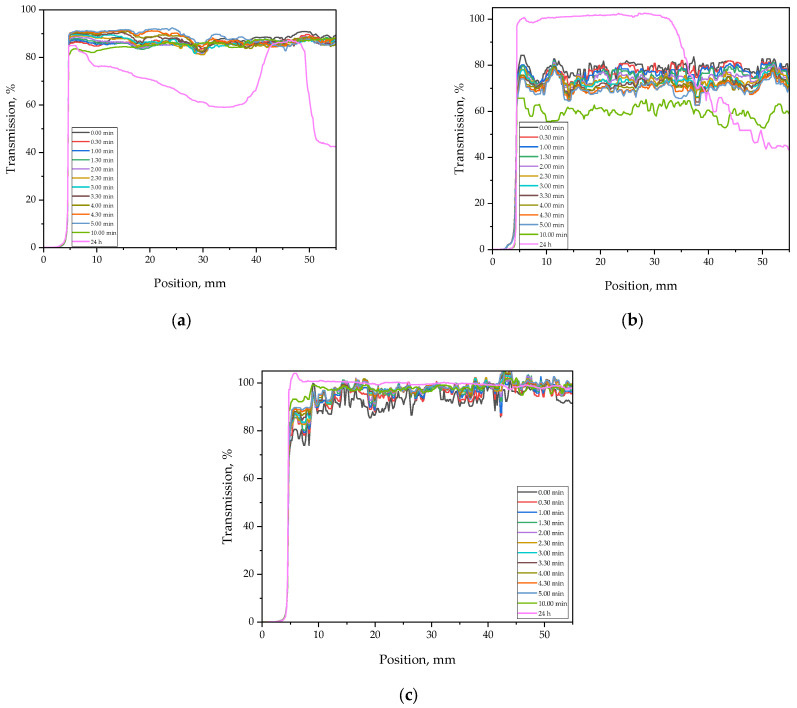
Stability of prepolymer mixtures measured at different time points before photopolymerization. From left to right: (**a**) 0SA (no sodium alginate), (**b**) 5SA (with 5 mL of 2.5% sodium alginate), (**c**) 10SA (with 10 mL of 2.5% sodium alginate).

Results of measurements obtained using the Multiscan system, showing time-dependent transmission characteristics of samples containing various quantities of sodium alginate, are presented in Figure 3. These results enabled assessment of the influence of sodium alginate on the homogeneity of the formulations and the stability of the hydrogel materials.

As shown in Figure 3, the samples were analyzed prior to the photopolymerization process—immediately after mixing the components, every thirty seconds over a period of 5 min, as well as after 10 min and 24 h from preparation. The initial transmission level was relatively high (approximately 80%) for all tested samples, indicating good homogeneity of the mixtures. In subsequent measurements, the transmission varied depending on the sample composition.

The transmission profile of Sample 10SA remained the most stable over time, which correlates with its appearance (Figure 2)—it was the most transparent of all tested samples. In this case, the transmission gradually increased after formulation preparation, possibly indicating improved clarity of the mixture and a reduction in light scattering phenomena [34].

The transmission profile of Sample 0SA_5 remained relatively stable during the first 10 min, with values around 80–85% across the entire measurement range (5–55 mm), which confirms good initial uniformity. However, after 24 h, a significant drop in transmission was observed—particularly between 5 mm and 35 mm—where values decreased to below 40% in some regions. Additionally, the profile exhibited irregular fluctuations (oscillations) in this zone, suggesting physical instability of the system, such as phase separation or sedimentation of components [34].

For the sample containing 5 mL of sodium alginate (5SA), the transmission also fluctuated around 70–80% during the first 5 min, but with notably greater curve dispersion compared to Sample 0SA. This may indicate less uniform dispersion of the components or the presence of small air inclusions within the system. After 10 min (green line), a decline in transmission was observed, and after 24 h (pink line), the curve revealed spatially heterogeneous destabilization: transmission initially increased in the 0–20 mm range, but then dropped sharply, reaching levels below 50%, and in some areas even lower. This behavior may suggest component migration or partial coagulation of hydrophilic substances, despite the presence of alginate. At this concentration (5 mL), sodium alginate likely does not provide sufficient viscosity or phase compatibility, leading to local structural disturbances within the prepolymer system [35].

The transmission analysis of samples prior to photopolymerization demonstrated that system stability strongly depends on the sodium alginate content. The absence or insufficient amount of alginate leads to time-dependent destabilization of the mixture, manifested by a decrease in transmission and signal fluctuations, which may negatively affect the homogeneity and final quality of the obtained hydrogels.

### 3.2. Findings from FT-IR Spectral Analysis of Hydrogels After Synthesis and Incubation

**Figure 4:**
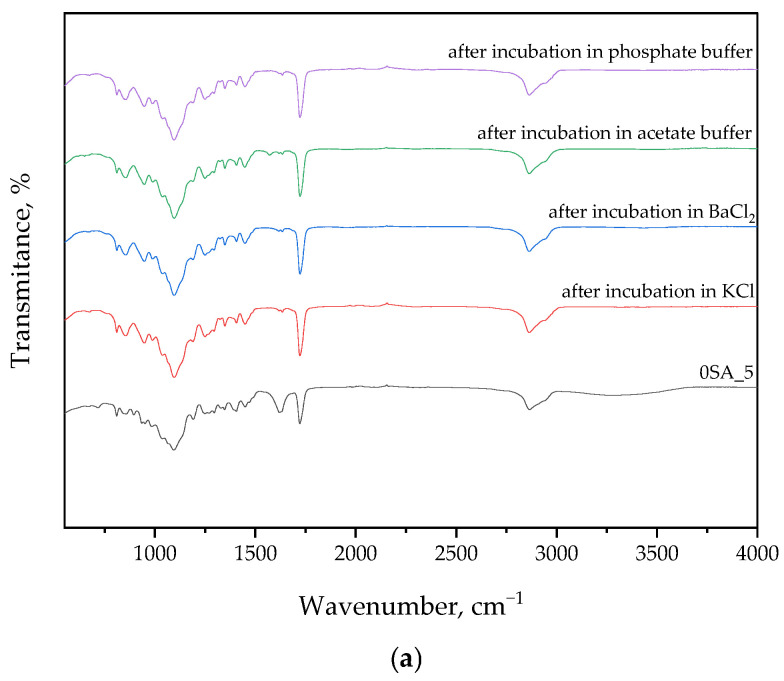

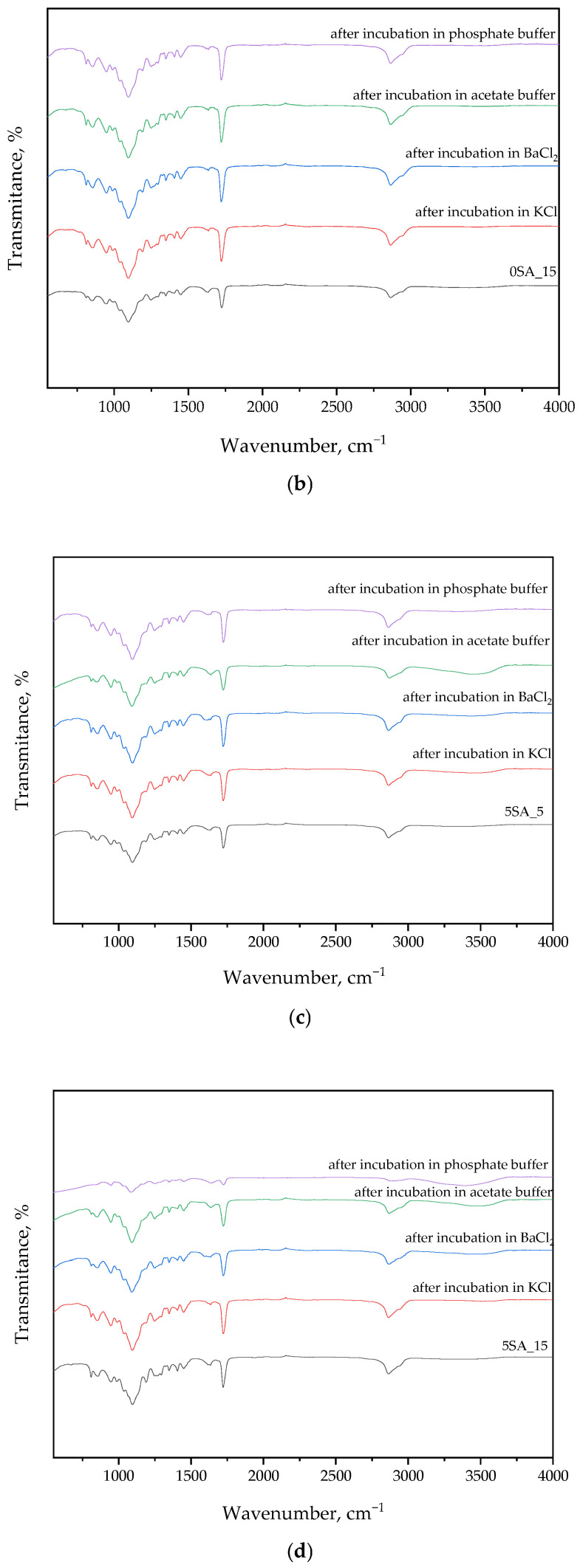

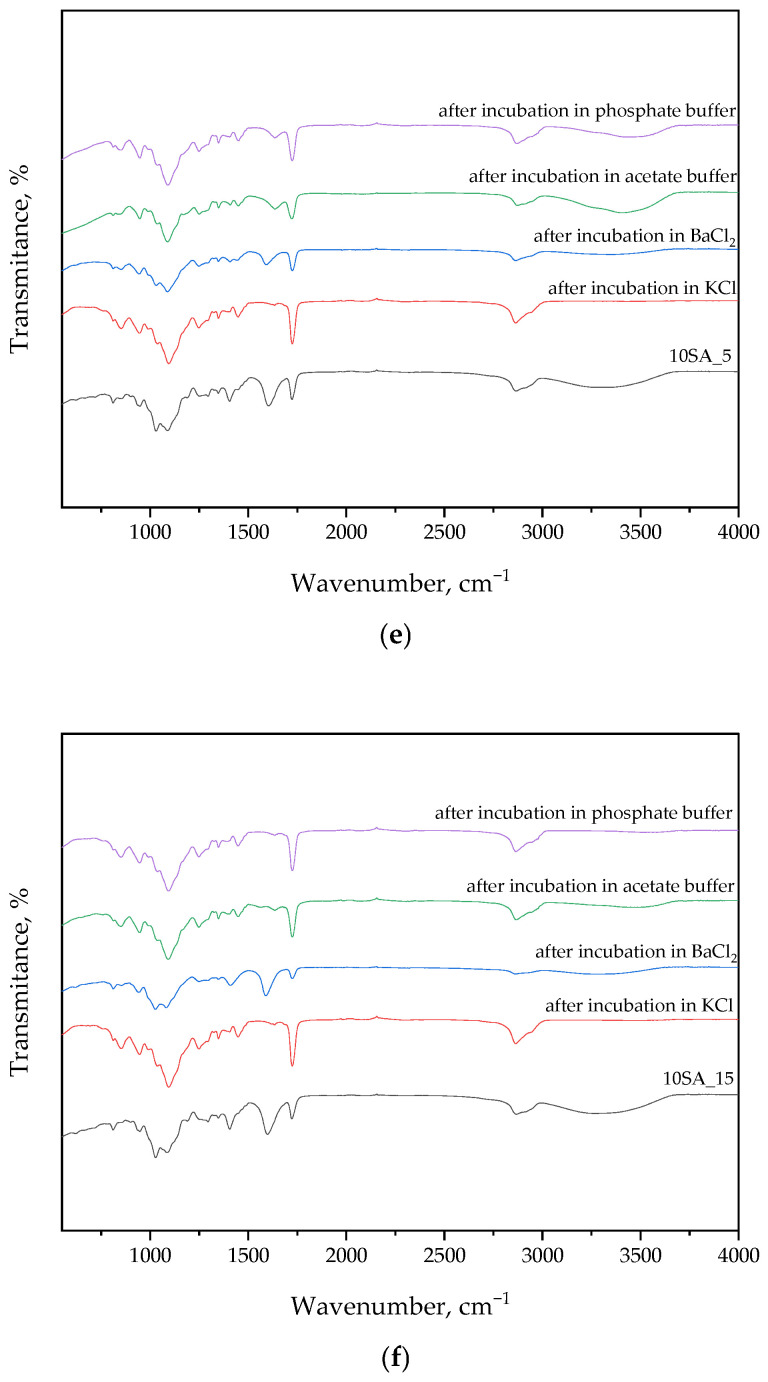
FT-IR spectra of hydrogels (**a**) 0SA_5, (**b**) 0SA_15, (**c**) 5SA_5, (**d**) 5SA_15, (**e**) 10SA_5, and (**f**) 10SA_15 before and after incubation in selected simulated physiological liquids.

The following section presents FT-IR spectral data, which were used to identify functional groups in the hydrogel matrix and to monitor potential chemical changes after incubation in various environments. The results are presented in Figure 4 and Table 2.

FT-IR analysis of samples before and after incubation in solutions generally showed no significant differences in the curves. This may indicate the absence of degradation during the incubation period and the preservation of the intact structure of the materials. Notably, more pronounced changes in peak intensities were observed for Sample 5SA_15 after incubation in phosphate buffer. This effect may result from ionic interactions between phosphate ions and carboxylate groups (–COO^−^) present in sodium alginate and gum arabic, leading to transient ionic crosslinking or conformational rearrangements. Such interactions may cause peak intensity modulation without altering the chemical structure, particularly in the regions corresponding to C=O, COO^−^, or OH stretching vibrations. Furthermore, phosphate-buffer-induced shifts in hydrogen bonding networks could slightly affect the vibrational energy of hydroxyl-containing groups, which is consistent with the observed spectral fluctuations see also [36].

**Table 2:** Absorption bands visible in FT-IR spectra with assigned functional groups originating from reagents used during the synthesis of hydrogels [35,37,38,39,40,41].

Wavenumber, cm^−1^	Functional Group	Substrate
3300	–OH	Betaine
1500	–N-H	
1400	–C-N	
1000	–CO	
3400	–OH	Gum Arabic
2900	–C-H	
1600	–COOH	
1400	–COOH	
1250	–CO –C-O –C-O-C	
1060	–C-H	
3600–3000	–OH	Sodium Alginate
2900	–C-H	
1600	–C=O	
1400	COO–	
1030	–C-O-C	
2885	–C-H	Crosslinking Agent (PEGDA)
1720	–C=O	
1200	–C-O-C	
3000	–C-H	Photoinitiator
1715	–C=O	
1600	–C=C	

As shown by FT-IR studies, betaine-derived peaks were observed in the spectrum, such as the band at 3300 cm^−1^, which corresponds to the –OH group. In addition, stretching vibrations were also recorded at 1500 cm^−1^, 1400 cm^−1^, and 1000 cm^−1^, which correspond to the N-H, C-N, and C-O bands [37,42].

Fourier-transform infrared (FT-IR) spectra also show peaks originating from gum arabic. A characteristic absorption band at 3400 cm^−1^ attributed to hydrogen bonds and stretching vibrations of –OH groups was observed. Bands at 2900 cm^−1^ indicate the presence of sugars, among others, as well as the presence of C-H alkane stretching. The peaks at 1600 cm^−1^ and 1400 cm^−1^ are attributed to asymmetric and symmetric stretching vibrations of carboxyl (COOH) groups. The band at 1250 cm^−1^ represents the stretching of C-O alcohol, C-O-C ether, and CO carboxylic acid. The band at about 1060 cm^−1^ represents the bending of the C-H alkene of gum arabic polysaccharides [38,39].

In the few samples’ FT-IR spectrum of sodium alginate, broad bands were observed in the range from 3600 to 3000 cm^−1^, which confirms the presence of –OH groups. Subsequently, a peak was observed at a wavenumber of 2900 cm^−1^, which is related to the oscillation band of the –CH alkyl groups. Next, the peak detected at 1600 cm^−1^ is characteristic of sodium alginate and indicates C=O stretching oscillations. The band at 1400 cm^−1^ wave number indicates oscillation of COO– groups, and this confirms the presence of carboxyl groups in the alginate molecule. And the peak at 1030 cm^−1^ indicates the presence of –COC groups [40,43].

Strong peaks derived from PEGDA were detected in FT-IR spectra. A peak near 2885 cm^−1^ generated by C-H bond stretching vibrations was recorded. Peaks at 1720 cm^−1^, corresponding to the C=O bond, and 1200 cm^−1^ for the C-O-C bond were also recorded [41].

Bands from the photoinitiator (2-hydroxy-2-methylpropiophenone) were detected. They were read at wavenumbers 3000 cm^−1^, 1715 cm^−1^, and 1600 cm^−1^, corresponding to the following functional groups: –C-H, –C=O, and –C=C [35].

The presence of well-defined characteristic bands in the FT-IR spectra before and after incubation confirms the structural integrity of the hydrogels and indicates no significant chemical changes, demonstrating their stability under simulated physiological conditions.

### 3.3. Assessment of Swelling Behavior of the Hydrogel Matrix

**Figure 5:**
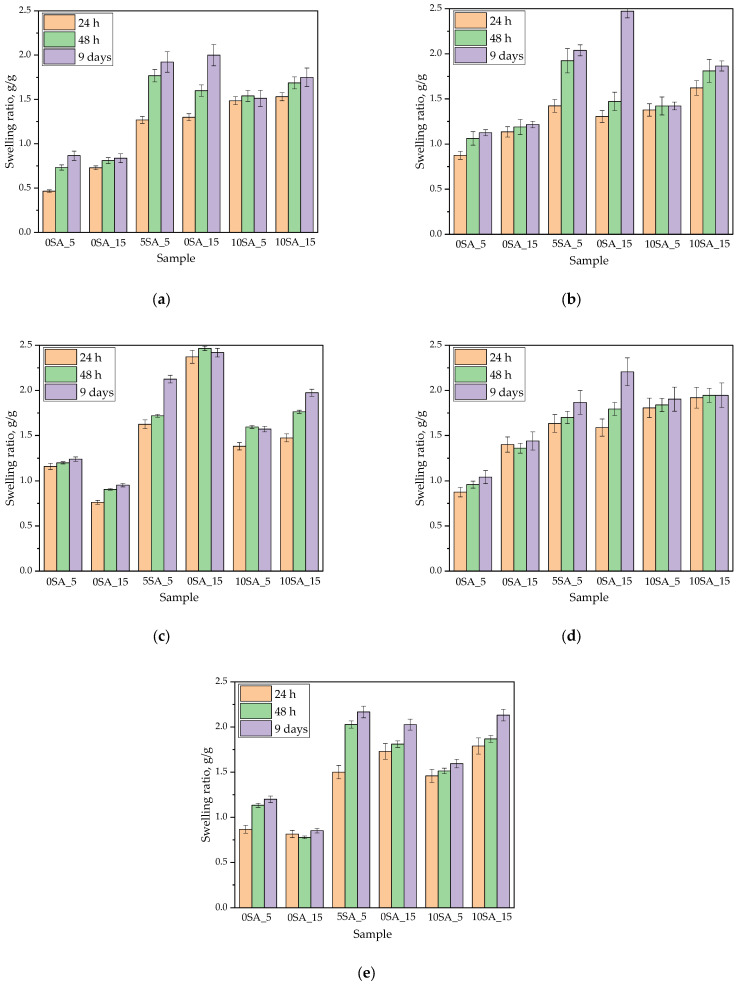
Swelling behaviors of the hydrogel samples after 24 h, 48 h, and 9 days of incubation in (**a**) phosphate buffer; (**b**) acetate buffer; (**c**) 0.9% potassium chloride solution; (**d**) 1 M barium chloride dihydrate solution; and (**e**) distilled water.

To evaluate the sorption properties of the synthesized hydrogels, swelling capacity was tested in various aqueous media. The results below illustrate how the composition of both the hydrogel and the external solution influenced swelling kinetics (Figure 5).

Swelling behavior was evaluated in five different aqueous media to assess the water absorption capacity and structural durability of the synthesized hydrogels. As shown in Figure 5, the swelling ratio varied significantly depending on the sample composition, incubation medium, and duration. The most pronounced differences were observed between formulations with different sodium alginate contents.

Hydrogels without sodium alginate (0SA_5 and 0SA_15) consistently exhibited the lowest swelling ratios across all incubation media. Their capacity to absorb liquid remained limited even after 9 days of incubation, typically not exceeding 1.5 g/g. This outcome is consistent with the results obtained from Multiscan analysis, which showed that these formulations were the most unstable prior to photopolymerization, displaying visible heterogeneity and phase separation. The absence of alginate likely reduced the hydrophilicity and elasticity of the polymer matrix, resulting in limited liquid uptake.

Conversely, samples containing the highest amount of alginate (10SA_5 and 10SA_15) demonstrated moderate swelling behavior with final values reaching approximately 1.8–2.0 g/g after 9 days. These samples maintained their structural integrity throughout incubation and did not exhibit visible degradation. Their performance suggests a balanced interaction between swelling capacity and mechanical stability, likely due to improved crosslinking efficiency and the stabilizing role of alginate in the polymer network.

Notably, the highest swelling ratios were observed for Samples 5SA_5 and 5SA_15, with values often exceeding 2.0 g/g across all media. However, despite these seemingly favorable results, both samples underwent visible disintegration during incubation, fragmenting into smaller pieces and losing their original shape. This fragmentation, although not reflected in the swelling data, significantly compromises their practical utility. The increase in surface area due to fragmentation likely contributed to the elevated swelling values, as smaller particles absorb water more readily. Therefore, these high values should not be interpreted as an indicator of hydrogel quality or structural performance.

Multiscan analysis supported these findings by revealing that Sample 5SA_5 was already unstable in the prepolymer state, showing a decrease in transmission over time and pronounced heterogeneity. FT-IR results indicated no major chemical degradation post-incubation, suggesting that the observed disintegration was mechanical in nature, driven by microstructural flaws rather than chemical breakdown. These findings suggest that two distinct degradation mechanisms may be involved. In the case of 5SA_5, insufficient crosslinking results in a loose polymer network with low cohesion, which cannot maintain its shape under osmotic pressure. In contrast, 5SA_15 likely exhibits excessive crosslinking density, forming a stiff and brittle structure that fractures when exposed to swelling-induced mechanical stress. This observation aligns with literature reports, where mechanical instability in PEGDA-based hydrogels has been linked to insufficient crosslinking density or low polymer chain entanglement, leading to fragile networks upon swelling [44]. In Sample 5SA_5, a shorter UV exposure time may have resulted in an under-crosslinked and loosely bound network, while in Sample 5SA_15, prolonged UV curing may have caused over-crosslinking and brittleness, making the matrix prone to fragmentation. Such contrasting effects of under- and over-crosslinking dynamics have also been observed in PEGDA systems, where swelling ratio alone does not guarantee mechanical integrity. Importantly, our hydrogels demonstrated swelling ratios ranging from approximately 1.0 to 2.5 g/g (with an average around 1.5 g/g), which is consistent with literature values for PEGDA hydrogels—typically in the range of ~1–3 g/g under similar experimental conditions [45].

In conclusion, while sodium alginate enhances the water absorption capacity of hydrogels, optimal performance requires a delicate balance between its concentration and UV curing conditions. Samples 5SA_5 and 5SA_15 highlight the risk of overinterpreting swelling data without considering structural integrity. Although they exhibited superior swelling performance numerically, their lack of mechanical stability renders them unsuitable for applications requiring long-term form retention, such as wound dressings or drug delivery matrices.

### 3.4. Results of the pH Analysis During Hydrogel Incubation

**Figure 6:**
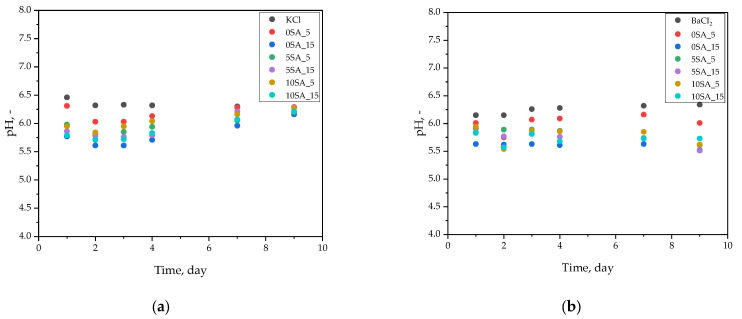

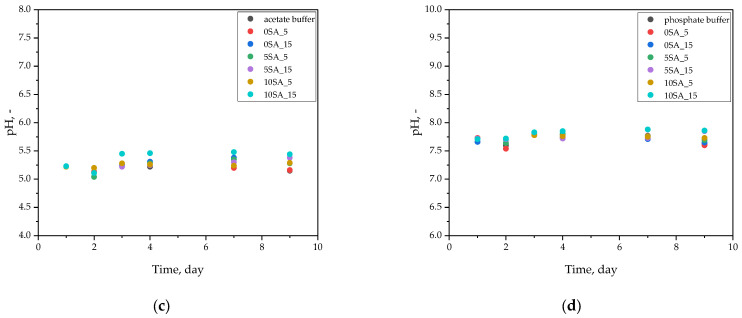
pH and temperature values of (**a**) KCl solution, (**b**) BaCl_2_ solution, (**c**) acetate buffer, and (**d**) phosphate buffer, measured during the hydrogels’ incubation period.

The interaction between hydrogel samples and incubation media was examined in terms of pH variation over time. The data (Figure 6) presented in this subsection reveal potential ion exchange or degradation effects during prolonged exposure to physiological fluids.

Throughout the 9-day incubation of hydrogel samples in various physiological solutions, both pH and temperature were monitored to assess environmental stability (Figure 6a–d). In general, only slight fluctuations in pH were observed, with most values remaining within ±0.3 units of their initial levels.

In 0.9% KCl solution (Figure 6a), a minor initial decrease in pH from ~6.2 to ~6.0 was observed, followed by stabilization at ~6.1–6.2, suggesting limited ionic interactions with the medium and a chemically inert hydrogel matrix.

In 1 M BaCl_2_ solution (Figure 6b), the pH remained consistently within the range of 5.6 to 6.3 throughout the incubation. This is noteworthy, as high concentrations of divalent cations like Ba^2+^ can potentially trigger ion exchange or additional crosslinking. However, the consistent pH indicates that the hydrogels were chemically resistant to such effects.

In acetate buffer (Figure 6c), an initial pH of ~5.2 gradually increased to ~5.4–5.5 over time. This mild shift could be attributed to limited ion exchange or buffering interactions with the hydrogel’s functional groups but did not affect the overall pH stability of the system.

The highest pH stability was recorded in phosphate buffer (Figure 6d), where values remained steady at 7.7–7.8 across all samples and timepoints. This is expected due to the strong buffering capacity of phosphate systems. Importantly, none of the samples caused measurable acidification or alkalinization of the medium, indicating that the hydrogels did not release significant amounts of ionizable species during the incubation.

As reported by Dilaver and Yurdakoc [46], hydrogel swelling may increase at higher pH values when the pH exceeds the pKa of functional groups, due to deprotonation and an increase in anionic charges in the network. However, the current data do not show notable pH changes that would suggest such an effect. This suggests the tested hydrogels were chemically stable, and their ionizable components remained well-buffered within each medium.

The study by Malektaj et al. [47] demonstrated that Ca-alginate–montmorillonite gels can maintain structural integrity across a wide pH range. Similarly, in the present study, samples such as 10SA_5 and 10SA_15 maintained both structural and pH stability, further confirming their robust formulation and mechanical resilience.

According to Manaila and Crăciun [48], stable pH values during incubation are indicative of a chemically stable hydrogel network. This observation aligns well with the present findings. However, mechanical disintegration observed in some samples (e.g., 5SA_15) despite stable pH values highlights an important point: chemical stability does not necessarily correlate with structural integrity.

### 3.5. Results of Contact Angle Measurements on Hydrogel Surface

To explore the surface characteristics of the hydrogels, contact angle measurements were conducted. The outcomes below reflect differences in surface wettability depending on UV polymerization time and material composition.

**Figure 7:**
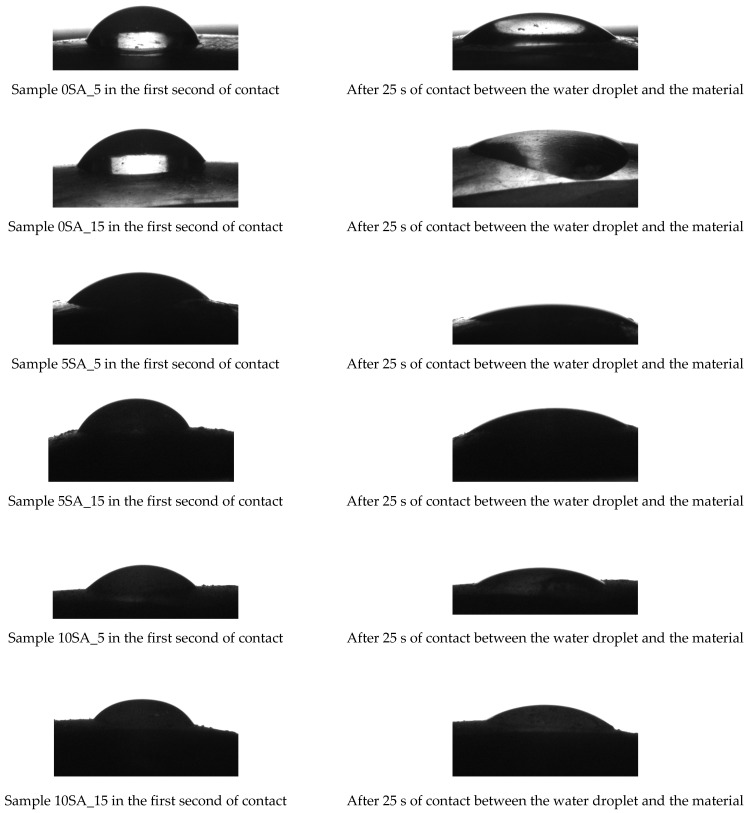
Contact angle images of hydrogel samples recorded using the Krüss DSA100M instrument. Each pair of images shows the shape of a water droplet at the moment of contact (**left**) and after 25 s (**right**).

To visualize the wettability dynamics of hydrogel surfaces, a series of contact angle images was captured at two time intervals: immediately upon droplet deposition and after 25 s of contact. This comparative approach enabled the assessment of water absorption and spreading behavior across all hydrogel formulations (see Figure 7).

**Figure 8:**
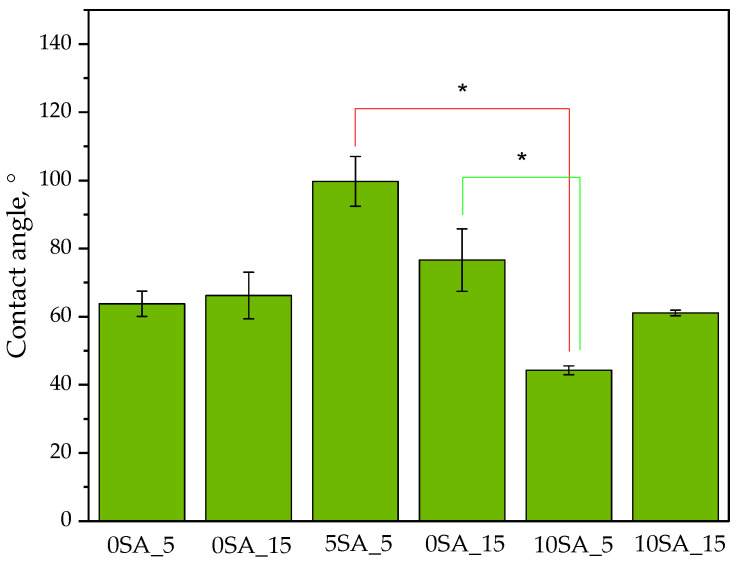
Contact angles of hydrogel samples, where water was used as the measuring liquid. Error bars represent standard deviations determined from three measurements. Statistical analysis was performed using two-way ANOVA, which revealed a significant effect of alginate content on surface wettability (*p* = 0.0179), while UV exposure time and interaction effects were not significant. Pairwise differences between selected groups were further analyzed using Welch’s *t*-tests; statistically significant differences (*p* < 0.05) are marked with *.

As shown in Figure 7, clear differences in droplet behavior were observed among the tested samples. Samples 0SA_5, 0SA_15, and 5SA_5, characterized by lower alginate content and/or shorter UV exposure time, exhibited rapid spreading and flattening of the water droplet over time, indicating higher surface wettability and likely greater porosity. In contrast, Samples 5SA_15, 10SA_5, and 10SA_15, with higher alginate concentrations or longer polymerization times, maintained a more defined droplet shape after 25 s of contact. This behavior suggests reduced wettability, possibly due to a denser crosslinked network or altered surface morphology. These findings indicate that both the alginate content and UV polymerization time play a critical role in tuning the hydrophilicity and surface characteristics of hydrogel materials. The contact angle measurements recorded at the first second of droplet contact offer insight into the initial surface wettability of the hydrogel samples, reflecting both their surface chemistry and morphology immediately after synthesis. Figure 8 presents the values of the determined wetting angles for the hydrogel samples. Water was used as the measuring liquid.

Measuring the contact angle provides insight into hydrogel behavior in humid environments and is an important parameter for evaluating materials intended for biomedical applications. As reported in the literature, lower contact angle values indicate higher surface wettability and greater hydrophilicity, which are desirable characteristics for drug delivery systems and wound dressings [49]. Conversely, higher contact angles suggest increased surface hydrophobicity and reduced initial interaction with aqueous media.

As shown in Figure 8, the contact angle values measured at the first second of water droplet contact varied significantly among the tested samples, depending on their chemical composition and UV exposure time during photopolymerization.

The highest contact angle was recorded for Sample 5SA_5 (~100°), indicating the lowest surface wettability among all the formulations. Interestingly, despite having the highest swelling ratio, this sample demonstrated limited initial water interaction at the surface. This may be attributed to a non-uniform or closed-pore surface morphology, potentially formed during polymerization, which could hinder immediate droplet absorption. This apparent contradiction—low surface wettability despite high swelling capacity—may be explained by the hydrogel’s microstructural properties. SEM analysis of 5SA_5 revealed a rough, cracked, and porous surface, which may limit immediate surface spreading of the droplet (thus increasing the contact angle), while simultaneously allowing internal capillary-driven water uptake after initial penetration. Such dual behavior has been described in other porous hydrogel systems, where macro-scale surface hydrophobicity coexists with rapid bulk water sorption due to internal porosity [50].

Samples 0SA_5 and 0SA_15 showed moderate contact angles (~65–70°), suggesting average wettability. The absence of sodium alginate may have contributed to a more porous and open surface structure, allowing for relatively quick droplet spreading despite the lack of hydrophilic alginate chains

The lowest contact angle (~50°) was observed for Sample 10SA_5, indicating the highest surface hydrophilicity. This could result from a balanced formulation with high alginate content and an optimal degree of crosslinking, providing uniform distribution of polar functional groups on the surface and enhancing water affinity.

Samples 5SA_15 and 10SA_15 displayed intermediate contact angles (~60–75°), implying that extended UV exposure might lead to denser crosslinking or a less accessible surface, thereby reducing wettability despite higher alginate content

These observations are consistent with reports by Holler et al. [51] and Kakarla et al. [52] where cross-linked alginate systems exhibited increased contact angles due to denser matrix formation. In contrast, our results suggest that alginate concentration plays a more decisive role in governing surface hydrophilicity than UV exposure alone.

To statistically validate these effects, a two-way ANOVA was performed. The analysis confirmed that alginate volume (0, 5, or 10 mL) had a statistically significant effect on contact angle values (*p* = 0.0179), while UV curing time and the interaction between factors were not significant (*p* > 0.05). Post-hoc Welch’s t-tests further showed that hydrogels with 10 mL of alginate exhibited significantly lower contact angles than those with 5 mL, confirming increased hydrophilicity. These results highlight the importance of formulation design—particularly alginate concentration—in tuning surface wettability, which is critical for ensuring desired interactions with biological tissues and fluids.

### 3.6. SEM-Based Evaluation of Hydrogel Structure

Scanning electron microscopy was employed to visualize the surface architecture of the hydrogel samples directly after synthesis. The images presented in the table below (Table 3) highlight the impact of formulation on the development of the porous polymer network and surface features.

The SEM images (Table 3) reveal clear differences in the surface morphology of the analyzed hydrogels depending on the sample composition and photopolymerization time. Contrary to the general claim that all samples exhibit an undulating surface, only some of them—particularly 5SA_5 and 5SA_15—display pronounced waviness, cracks, and porous regions. Their surfaces appear visibly rough and irregular, which suggests looser crosslinking and potentially greater accessibility to water, correlating with their high swelling ratios.

In contrast, Samples 0SA_5 and 0SA_15 show relatively smooth and compact surfaces, with few indentations or visible pores. This indicates a denser polymer network structure and lower capacity for water uptake. Their morphology appears more homogeneous, but also less reactive in terms of surface interaction with aqueous environments.

Samples 10SA_5 and 10SA_15 exhibit an intermediate surface morphology—not as smooth as the 0SA samples, yet lacking the prominent undulating structures seen in 5SA_5 and 5SA_15. This suggests a more uniform and stable crosslinked network with moderate porosity, which corresponds to their balanced swelling behavior and high structural integrity during incubation.

The irregular bright features visible on some surfaces are most likely surface artifacts (e.g., micro-contaminants, fractures, or shrinkage effects) rather than clearly identifiable unbound particles. According to Martínez-García et al. [53], the swelling behavior of hydrogels strongly depends on surface structure and porosity, which are in turn influenced by the concentration of the applied polymer and the crosslinking conditions. The SEM findings in this study support that observation—samples with higher porosity (5SA series) exhibited the greatest swelling, while those with smoother surfaces (0SA and partially 10SA) showed reduced absorbency but maintained greater mechanical integrity.

Overall, the SEM analysis confirms that hydrogel surface morphology is strongly influenced by sodium alginate content and photopolymerization parameters. Samples with highly porous and irregular structures (5SA) demonstrated enhanced swelling capacity but lower mechanical stability. In contrast, smoother-surfaced samples (0SA) were more structurally stable but less absorbent. These findings reinforce previous results on the sorption behavior and long-term stability of the materials during incubation. These morphological differences also help explain the observed discrepancies between surface wettability and swelling performance—for instance, in Sample 5SA_5, the combination of rough surface topography and internal porosity likely limited droplet spreading (high contact angle) while still enabling significant water uptake through capillary action.

### 3.7. Surface Morphology Evaluation Using Optical Microscopy

**Figure 9:**
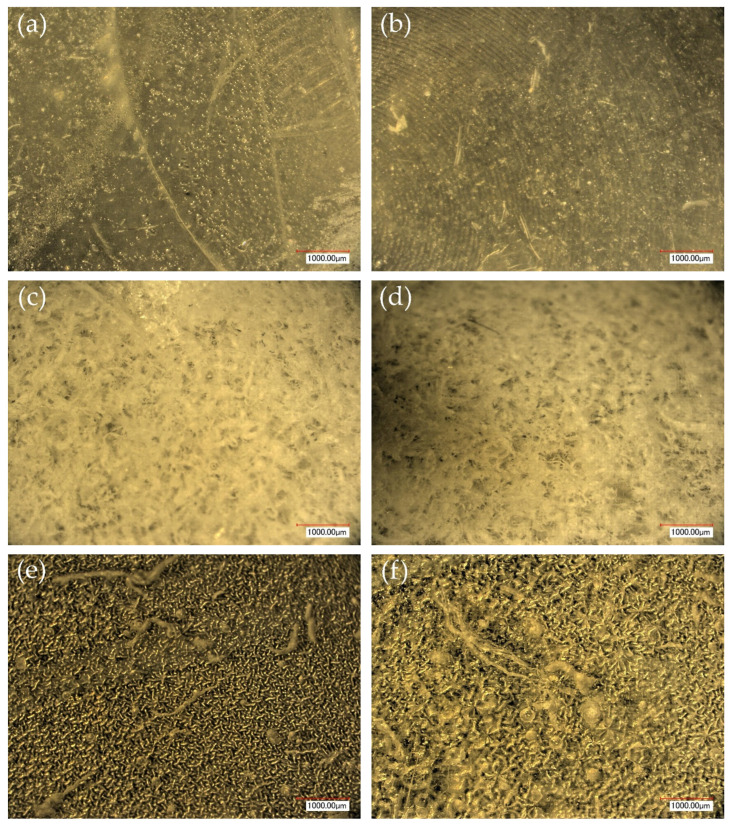
Surface morphology images of hydrogel samples: (**a**) 0SA_5, (**b**) 0SA_15, (**c**) 5SA_5, (**d**) 5SA_15, (**e**) 10SA_5, and (**f**) 10SA_15.

In order to obtain complementary information on surface characteristics, high-resolution profilometric analysis was conducted using a 4K digital microscope. The results below show surface texture variations between hydrogel samples, with particular attention to the influence of UV polymerization time (Figure 9).

The optical microscopy images (Figure 9) support and complement the SEM observations, clearly demonstrating the influence of both sodium alginate content and UV polymerization time on the surface structure of the hydrogels.

Samples without alginate (0SA_5 and 0SA_15) appear relatively smooth and homogeneous, in agreement with SEM results, indicating a compact, low-porosity network. The addition of 5 mL of alginate (5SA_5 and 5SA_15) results in visible irregularities, rougher texture, and emerging pores, while 10 mL (10SA_5 and 10SA_15) leads to a markedly more undulating and complex surface. These changes reflect increasing surface roughness, which correlates with higher swelling capacity but also reduced mechanical stability.

Notably, extended UV curing time (15 min) further accentuates surface irregularities—these samples exhibit more pronounced granularity and microstructural complexity compared to their 5 min counterparts, which is consistent with SEM findings.

These results align with the observations of Korzhikov-Vlakh et al. [54], who reported that incorporating alginate into polymer matrices increases surface roughness. In the present study, higher alginate concentrations and prolonged UV exposure led to more developed surface textures, which may influence biological interactions and adhesion properties of the hydrogels.

Additionally, the ring-like patterns visible in some samples (e.g., 0SA_5 and 0SA_15) are not biological contaminants such as fingerprints, but rather surface artifacts, likely resulting from the interaction between the prepolymer mixture and the casting substrate during UV curing. These features were only observed in selected formulations and did not affect the overall morphology-related interpretation.

## 4. Conclusions

The conducted study demonstrated that both the composition of the hydrogel formulations and the UV polymerization time significantly influenced the structural, sorption, and surface properties of the resulting materials. Increasing the sodium alginate content from 0% to 10% markedly improved the homogeneity and stability of the prepolymer mixtures, as confirmed by Multiscan transmission profiles. Samples containing 10% alginate (10SA_5 and 10SA_15) maintained stable optical transmission above 80% even after 24 h, indicating excellent dispersion and phase compatibility.

The swelling capacity varied widely depending on formulation. Although the highest swelling ratio was observed for the 5SA_5 sample (2.32 g/g after 9 days in distilled water), this formulation suffered from mechanical disintegration during incubation, making it unsuitable for biomedical use where form stability is required. In contrast, the 10SA_15 sample exhibited a balanced performance, achieving a moderate swelling ratio of 1.92 g/g while maintaining structural integrity over the entire incubation period.

FT-IR analysis confirmed the chemical stability of all formulations in simulated physiological fluids, with no significant shifts in peak positions or intensities, indicating no degradation of functional groups. The sample 5SA_15 showed some spectral intensity changes after incubation in phosphate buffer, potentially due to minor structural rearrangements, yet these did not affect the core chemical integrity of the hydrogel matrix.

Surface wettability, evaluated via contact angle measurements, ranged from 50° to 100°. The lowest value, indicating highest surface hydrophilicity, was recorded for the 10SA_5 sample, suggesting its suitability for applications requiring rapid fluid interaction, such as wound dressings or biosensors. In contrast, 5SA_5, despite its high swelling ratio, exhibited a contact angle of 100°, pointing to a possible closed-pore or uneven surface that delayed droplet absorption.

Microscopic observations revealed that the surface morphology was strongly influenced by alginate content and UV exposure time. SEM and optical microscopy showed that samples with 0% alginate had smooth, dense surfaces and limited porosity, while 5SA samples displayed highly porous, cracked structures correlating with their high swelling but poor mechanical resistance. The 10SA series presented the most desirable morphology—moderately porous and structurally uniform—aligning with their superior balance of swelling behavior and mechanical robustness.

In conclusion, hydrogel formulations containing 10% sodium alginate and polymerized for 15 min (10SA_15) offered the best compromise between swelling capacity (1.92 g/g), surface hydrophilicity (contact angle~60°), and long-term structural integrity, making them promising candidates for further development in biomedical applications such as absorbent wound dressings or drug delivery systems.

Limitations and future work: While the developed hydrogel systems demonstrate promising physicochemical properties, their practical application in bioanalytical or biomedical platforms remains limited at this stage. In particular, the mechanical disintegration observed in certain formulations (e.g., 5SA_5 and 5SA_15) reduces their utility for applications requiring long-term shape stability, such as microneedle arrays or wound dressings. Additionally, the current materials lack functional surface groups needed for the stable immobilization of biomolecules. Future studies will focus on improving structural robustness, introducing surface functionalities, and evaluating biocompatibility to assess their potential for integration into bioanalytical microdevices and drug delivery systems.

## Figures and Tables

**Table 1:** Composition of hydrogel samples used for photopolymerization.

Sample	Betaine 5%	Gum Arabic 5%	Sodium Alginate 2.5%	Crosslinking Agent	Photoinitiator	UV Polymerization Time
	[mL]	[mL]	[mL]	[mL]	[mL]	[min]
0SA_5	2	2	0	7	0.25	5
0SA_15			0			15
5SA_5			5			5
5SA_15			5			15
10SA_5			10			5
10SA_15			10			15

**Table 3:** Images of hydrogels at different magnifications, captured using SEM.

Sample	SEM Images Taken at Different Magnifications		
0SA_5	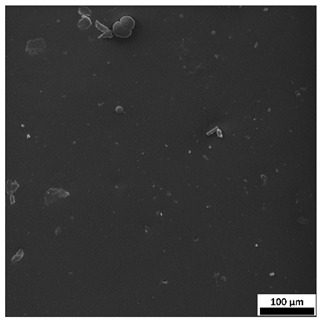	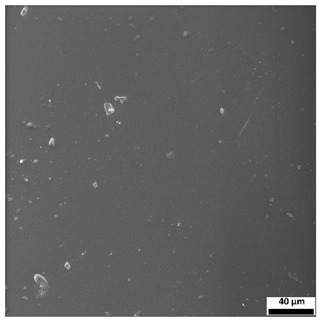	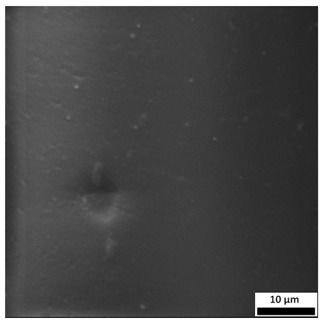
0SA_15	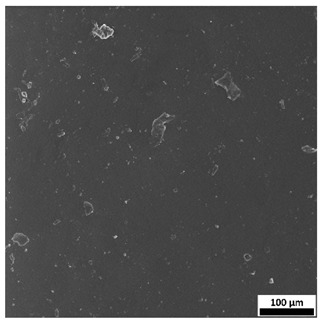	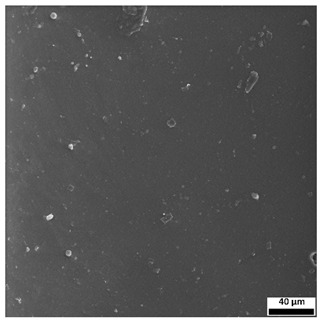	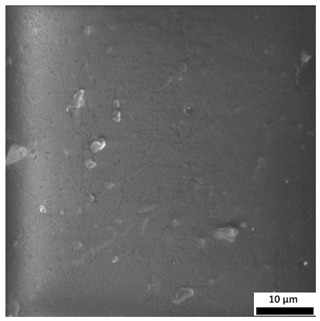
5SA_5	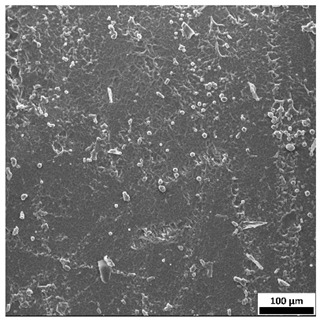	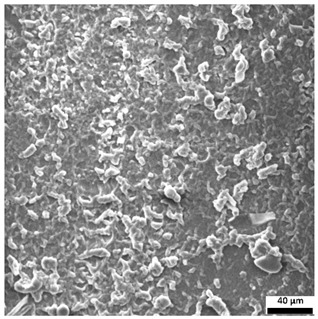	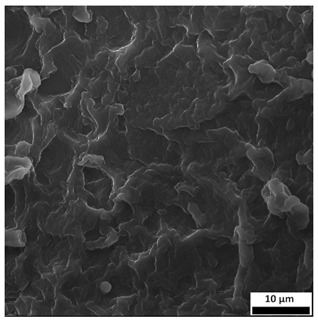
5SA_15	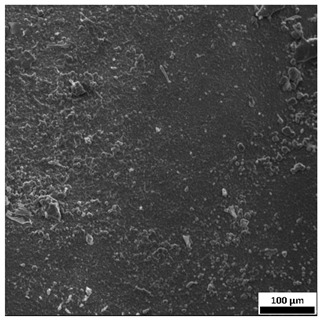	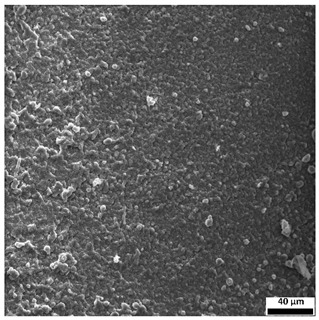	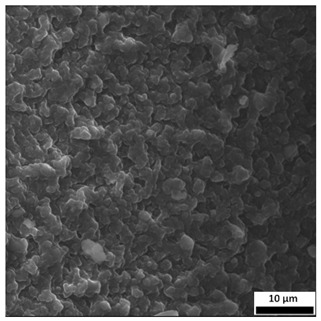
10SA_5	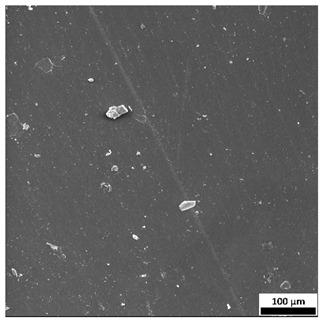	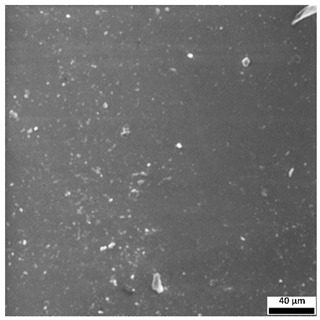	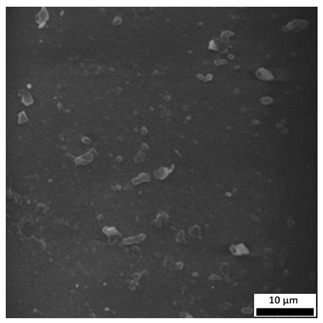
10SA_15	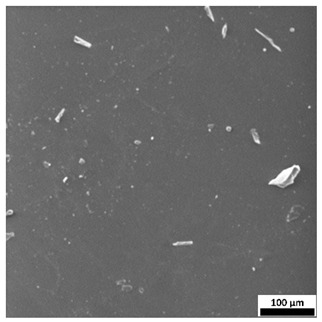	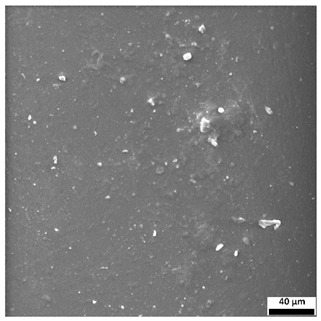	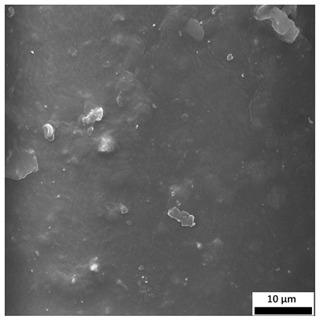

## Data Availability

The original contributions presented in this study are included in the article. Further inquiries can be directed to the corresponding authors.
